# Isolation, Purification, Characterization and Effect upon HepG2 Cells of Anemaran from *Rhizome Anemarrhena *

**Published:** 2013

**Authors:** Qian-Qian Jiang, Yun-Ping Zhao, Wen-Yuan Gao, Xia Li, Lu-Qi Huang, Pei-Gen Xiao

**Affiliations:** a *Tianjin Key Laboratory for Modern Drug Delivery and High-Efficiency, School of Pharmaceutical Science and Technology, Tianjin University, Tianjin 300072, China. *; b *School of Chinese Medicine, Tianjin University of Traditional Chinese Medicine, 300193, China. *; c *Institute of Chinese Matetria Medica, China Academy of Chinese Medicinal Sciences, Beijing 100700, China. *; d *Institute of Medicinal Plant, Chinese Academy of Medical Sciences and Peking Union Medical College, Beijing 100094, China. *

**Keywords:** Anemaran, Isolation, Purification, Characterization, Hypoglycemic effect

## Abstract

The rhizome of *Anemarrhena asphodeloides *is used as food and traditional Chinese medicine for its hypoglycemic effect. The aim of this study was to investigate the isolation, purification and hypoglycemic activity of Anemaran as the active component. The influence factors (isolation duration, ratio of residuals to water and extracting times) during the isolation process were evaluated. The optimal conditions for NA and AA were extraction temperature 90ºC and 100ºC, duration 1h and 1.5 h, extraction time 3 and 3, and the solid–liquor ratio 1:20 and 1:15, respectively. Neutral and acid Anemaran (NA and AA) were isolated from the rhizome of *Anemarrhena asphodeloides*. Five fractions of NA-1, NA-2, NA-3, AA-1 and AA-2 were obtained after crude neutral and acid Anemaran purified through DEAE- 52 cellulose anion-exchange column. The characterizations of Anemaran and its different fractions were both analyzed by Fourier transform infrared spectroscopy (FT-IR) and scanning electron micrographs (SEM). Structural properties of different fractions were examined by FT-IR. Strong characteristic absorption peaks were observed at around 1744 cm^−1^and 1650 cm^−1^ caused by the C=O group of uronic acids, and the band between 1440 cm^−1^ and 1395 cm^−1^ associated with the stretching vibration of C–O of galacturonic acid. Neither the crude neutral, nor the acid anemaran significantly inhibited the growth of HepG2 cells *in-vitro*, which indicated the low cytotoxicity of the anemaran. Furthermore, both neutral and acid anemaran showed hypoglycemic effect. The hypoglycemic effect of neutral anemaran was much higher than that of acid anemaran.

## Introduction

The rhizome of *Anemarrhena asphodeloides *Bunge (Liliaceae) has been widely used as a traditional Chinese medicine. Its biochemical composition mainly consists of proteins, carbohydrates, steroidal saponins, flavonoids, xanthenes and norlignans. It has been reported that Rhizome Anemarrhenae has the activities of anti-diabetes (1, 2) and diureses (3). The steroidal saponins (4, 5), xanthenes (6) and norlignans (7) were major small molecular constituents of Rhizome Anemarrhena. However, polysaccharide of *Anemarrhena asphodeloides *Bunge (Anemarans) has not been received enough attention. It was reported that some extracts of Rhizoma Anemarrhenae markedly inhibited growth of cancer cells directly, showed the activities of anti-diabetes, diureses and hypoglycemic effect (1-3, 8-9).

Diabetes is a health endangering chronic metabolic hereditary disease with hyperglycaemia. It is characterized by metabolic disorders accompanied with hyperphagia, a selective loss of pancreatic islet β cell mass that results in absolute or relative deficiency of insulin secretion. Impaired insulin action threats human health. The advanced treatments mainly focus on injecting insulin, oral hypoglycemic drugs and so on. However, these drugs may cause side effects to varying degrees. Therefore, more and more researches were to make drugs with high efficiency and low toxicity, particularly the drug extracted from natural resources with hypoglycemic activity and little side effects (10-12). Therefore, much attention should be paid on component from plants.

Anemarans (AM) are the components in Rhizome Anemarrhena and comprise up to 20% of the rhizome. There are various reports about immunological activity of the polysaccharide from *Dioscorea opposita *Thunb. roots, antitumor activity of sulfated polysaccharide from *Gynostemma pentaphyllum *Makino, hypoglycemic effect of crude exopolysaccharides of *Phellinus baumii*, *in-vitro *antitumor and antioxidant activities of polysaccharide from *Sargassum pallidum *(13-15). It was also reported that neutral anemarans A, B, C and D showed hypoglycemic effect *in-vivo *(1, 16). Due to its various activities mentioned above, polysaccharide has attracted great attention. In order to obtain these functional components, numerous studies were carried out further to isolate and characterize polysaccharides from plant and microbial sources with emergence of separation technologies, for example, DEAE-cellulose anion-exchange chromatography (DEAE 52-cellulose) for fractionating the extracts, high performance liquid chromatography for determining the molecular weight of each sub-fraction, and gas-liquid chromatography for analyzing neutral sugar composition of polysaccharides (17). 

In this study, we isolated purified polysaccharides from Rhizome Anemarrhena by hot water and cellulose *DEAE *52 anion-exchange column in order to purify fractions. Anemarans was analyzed the morphology characterization, IR characters of different anemaran fractions, and evaluated cell growth inhibition and hypoglycemic effect upon HepG2 cells *in-vitro*.

## Experimental

Rhizome Anemarrhena herbs were collected in April 2009 from its natural habitat in Anguo (Hebei, China) and authenticated by Professor Wen-Yuan Gao. A voucher specimen has been deposited in School of Pharmaceutical Science and Technology, Tianjin University (Tianjin, China) 

All the chemical reagents used were analytical grade and obtained from Guangfu Chemical Company (Tianjin, China). *DEAE *cellulose 52 anion-exchange column was obtained from Guoyao Company (China). 

The human hepatoma cell line, HepG_2_, was purchased from Cell Resource Center, Shanghai Institutes for Biological Sciences, Chinese Academy of Sciences.

*Extraction of neutral anemaran (NA) and acid anemaran (AA)*

100 g of the Rhizome Anemarrhena was washed, dried and comminuted to powders with a plant micro-muller and sieved with 160 mesh sifter. The dried powder (1:10, w/v) was extracted with 80% ethanol three times. Then the residuals were dried in baking oven and extracted with water for the desired duration (0.5 to 2.5 h) at the temperature of 60 ºC to 100 ºC. The ratio of residuals:water varied from 1: 5 to 1: 30. This procedure was repeated 1-5 times. The water-extracts were combined and centrifuged. The supernatant obtained was concentrated in a rotary evaporator, and then, precipitated with 95% ethanol (1:3, v/v) at 4 °C for overnight (17). The precipitation was deproteinated with absolute alcohol, acetone and ethyl ether successively washing, and then lyophilized to obtain about 20 g of crude neutral anemaran (NA).

The dried residuals (47 g) after extracted with water, were extracted with 0.1 mol/L NaOH aqueous solution for the extraction desired duration (0.5 to 2.5 h) at the temperature of 60ºC to 100ºC. The ratio of residuals: water varied from 1: 5 to 1: 30. This procedure was repeated 1-5 times. The water-extracts were combined and centrifuged. The supernatant obtained was concentrated in a rotary evaporator, and then, precipitated with 95% ethanol for overnight (1: 3, v/v) at 4 °C. The supernatant was deproteinated with absolute alcohol, acetone and ethyl ether. The supernatant was lyophilized to give about 1.25 g of crude acid anemaran (AA). (18-20).

*Purification of polysaccharide*

500 mg of the dried powders of crude polysaccharides (NA and AA) were redissolved in deionized water and forced through a filter (0.45 μm), then applied to a column (2.6 × 50 cm) of DEAE-Cellulose A52. After loading with sample, the fractions was eluted with gradient NaCl aqueous solution (0, 0.1, 0.3 and 0.5 mol/L) at a flow rate of 9 mL/h. Fractions of 5 mL were collected and monitored by the phenol-sulfuric acid method at 490 nm, using glucose as standard (22, 23). Five different polysaccharide fractions, coded NA-1, NA-2, NA-3, AA-1 and AA-2 were obtained.

*Fourier transform infrared (FT-IR) spectroscopy*

FT-IR spectra of the samples were recorded with an IR spectrometer (Bruker Tensor 27) using potassium bromide (KBr) disks prepared from powdered samples mixed with dry KBr in a ratio of 1:100 in the frequency range 4000~400 cm^−1^.

*Scanning electron microscope (SEM)*

The anemarans and their purified fractions were detected by a scanning electron microscope (ESEM Philips XL-30) for morphological features. The dried samples that passed 200 meshes sieve, were fixed on a glass plate then gold powder was spread uniformly to make the sample conductive for the determination. An accelerating potential of 20kV was used during micrography. 

*Growth inhibition of HepG*_2_ *cells*

The inhibition effects of anemarans on the growth of HepG_2_ cells were evaluated *in-vitro *by the 3-(4, 5-dimethylthiazol-2-yl)-2, 5-diphenyltetrazolium bromide (MTT) dye reduction assay (M8180, Solarbio, China). Briefly, the HepG_2 _cells were seeded at a density of 1.0×10^5^ cells/ml into 96-well plates in a culture RPMI 1640 medium containing 10% FBS and in standard incubator conditions (37ºC, 5% CO_2_). After 24 h, the cultures were washed and treated with the absence or presence of 100 μL of anemarans (1 mg/mL, 0.5 mg/mL, 0.1 mg/mL and 0.05 mg/mL) for 24 h. And then, 10 μL of MTT solution (5 mg/mL) was added to each well and incubated for 4 h. 100 μL/well of DMSO were added to stop the reaction. The absorbance at 490 nm was measured using ELISA reader (Bio-Tek EL 800, USA) after culture medium was removed (21).

Growth inhibition rate (%) = (1−Absorbance of experimental group/Absorbance of blank control group) × 100%. 

*Glucose consumption assay*

Glucose consumption assay was used for determining hypoglycemic effect. HepG_2 _cells, the human hepatocarcinoma cell line were seeded in 96-well microplate with density of 10^5^ cells per well and maintained in RPMI 1640 medium containing 10% fetal calf serum with 5% CO_2_ at 37 ºC overnight before treatment. Subsequently, HepG_2_ cells were incubated with or without insulin (2×10^-6^ mol/L) for 16 h, washed once with serum-free medium for 20 min, and then treated with the polysaccharide fractions at different concentrations (1, 0.5, 0.1 and 0.05 mg/mL) in the presence of 2×10^-6^ mol/L insulin for 24 h (15, 16). The glucose concentrations in cell culture supernatant of each group were determined by Glucose assay kit at 505 nm (Rongsheng, Shanghai) following the manufacturer’s instruction. Hypoglycemic effect was expressed as glucose consumption.

*Statistical analysis*

All assays were carried out in triplicates and the results were expressed as mean ± SD. Statistical analysis was performed using one-way ANOVA followed by Duncan multiple comparison test according to the SPSS 17.0 system. A probability of p < 0.05 was considered significant. 

## Results and Discussion

*Optimization for extraction of neutral and acid anemaran*

**Figure 1:**
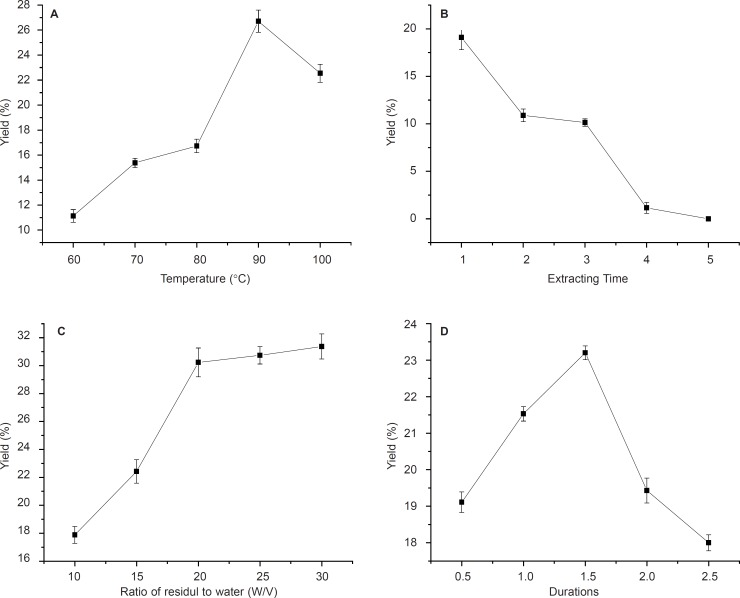
Effects of temperature, time of extraction, ratio of residual water and reaction durations on NA yield. Results were presented as means ± SD (n = 3).

**Figure 2:**
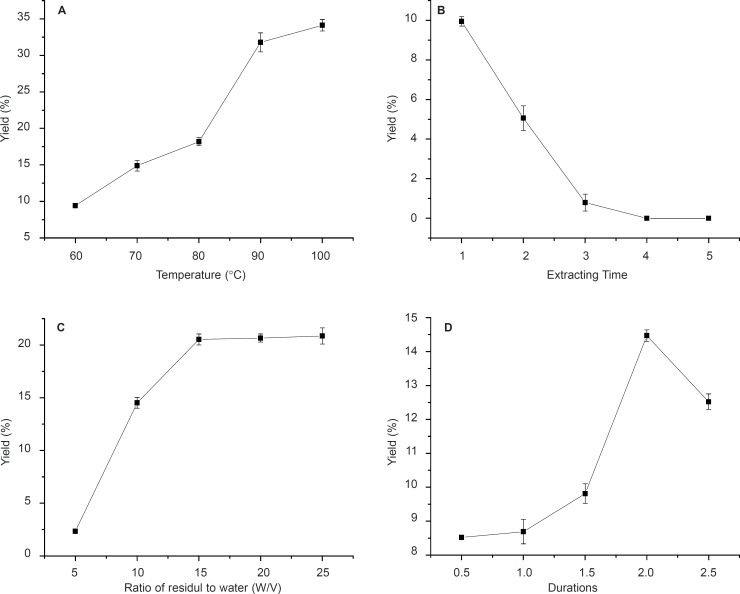
Effects of temperature, time of extraction, ratio of residual water and reaction durations on AA yield. Results were presented as means ± SD (n = 3).

The effect of variation of four parameters, isolation duration, temperature, the ratio of residuals to water and extracting times on neutral and acid polysaccharide yields were investigated in this study (Figure 1, 2). 

The effects of different temperatures of 60, 70, 80, 90 and 100ºC on polysaccharides (NA and AA) yields are shown in Figure 1a and Figure 2a. With temperature increasing, the yield of crude polysaccharides increased until 90ºC for NA (Figure 1a). Therefore, 90ºC was selected as the centre point of extracting temperature in this experiment as higher temperature would cause energy waste and decrease the yield. This could be due to the fact that higher temperature might cause degradation of anemaran (24, 25). However, it is observed that the yield of AA increased from 9.2% to 32% with the temperature increasing from 60 to 100 ºC. The yield of polysaccharides obtained at 90 ºC was significantly higher than that obtained at 60, 70 and 80ºC, but not significantly different from that obtained at the temperature of 100ºC for AA (Figure 2a). The optimum temperature at 90 ºC was good for both producing more NA and AA. The results of extraction times affecting on the yield of polysaccharides are shown in Figure 1b and Figure 2b. 

The results obtained by investigating the effect of solid and liquor ratio on DS are shown in Figure 1c and Figure 2c. The amount of residuals and reactants used during these experiments was constant, but the ratio of residuals/water decreased by increasing the amount of solvent. The optimum yield at residuals/water ratio was 1: 20 for NA and 1:15 for AA, at the ratio of which the most of polysaccharide was isolation (18, 19). 

The polysaccharide yield of the fourth time extraction became very low compared with three times. In order to improve production efficiency of NA and AA, this process could be held on for three times. The yield increases with the extraction durations elevated and reaches a maximum in 1.5 h for NA and a significant decrease was observed when the time increasing thereafter. Similar observations were observed in 2h for AA (Figure 1d and Figure 2d). 

*Purification of polysaccharide*

**Figure 3:**
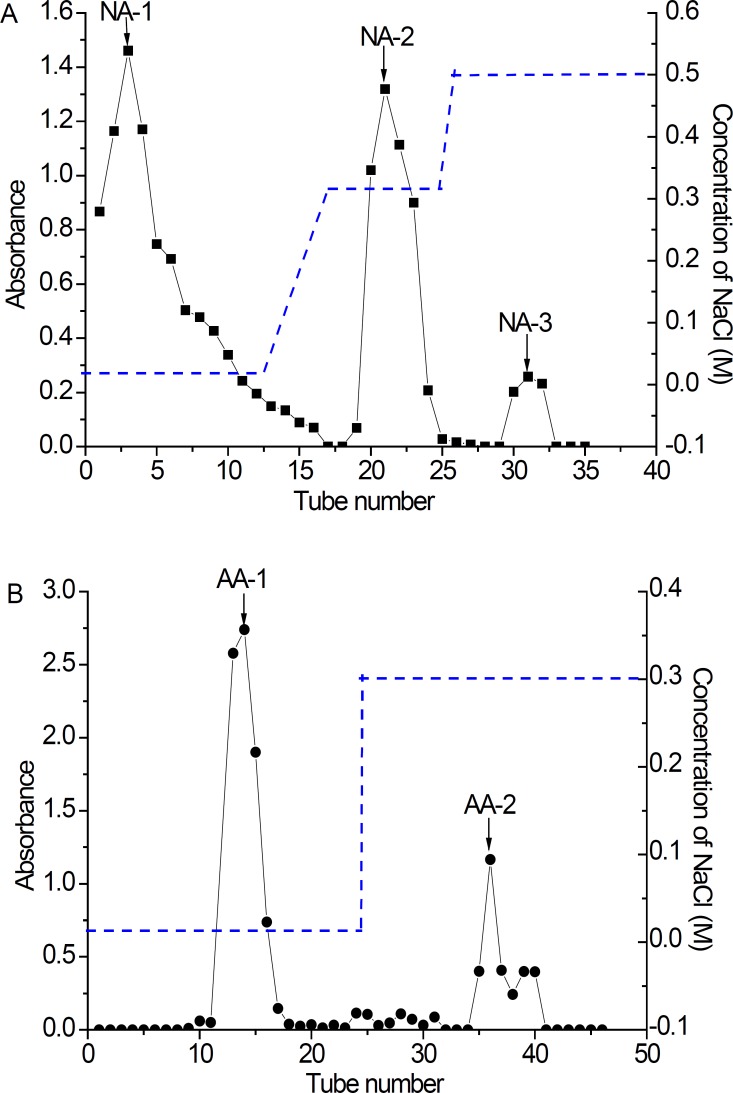
Elution curve of polysaccharide fractions in DEAE–cellulose 52 anion-exchange column purified by different concentrations of NaCl aq. A: NA (1, 2 and 3); B: AA (1 and 2) (-●- Absorbance at 490 nm .... NaCl gradient). NA: Neutral Anemaran; AA: Acid Anemaran.

The extracts of NA and AA were fractionated by DEAE-cellulose-52 anion-exchange column to obtain five main fractions (NA-1, NA-2, NA-3, AA-1 and AA-2) based on total carbohydrate elution profile (Figure 3). All the fractions of NA and AA appeared as white powders. They had no absorption at 280 or 260 nm in the UV spectrum, indicating the absence of protein and nucleic acid. After NA fractionation on DEAE– cellulose 52 anion-exchange column, NA-1 (166.9 mg), NA-2 (100 mg) and NA-3 (16.8 mg) were obtained from 0 mg/L, 0.1 mg/L and 0.3 mg/L NaCl elution, respectively (Figure 3a). In a similar manner, a lyophilized fraction of polysaccharides AA was chromatographed on a DEAE Cellulose-52 anion-exchange column to yield two peaks, AA-1 (98.2 mg) and AA-2 (180 mg) (Figure 3b). However, there were no absorbance for 0.1 mol/L NaCl elution of NA, and 0.1 and 0.5 mol/L NaCl elution of AA, respectively. It is reported that the concentration of sample, elution rate and exchange capacity of the chromatography column was closely related to separation effect (14, 18). 

*FT-IR analysis*

**Figure 4:**
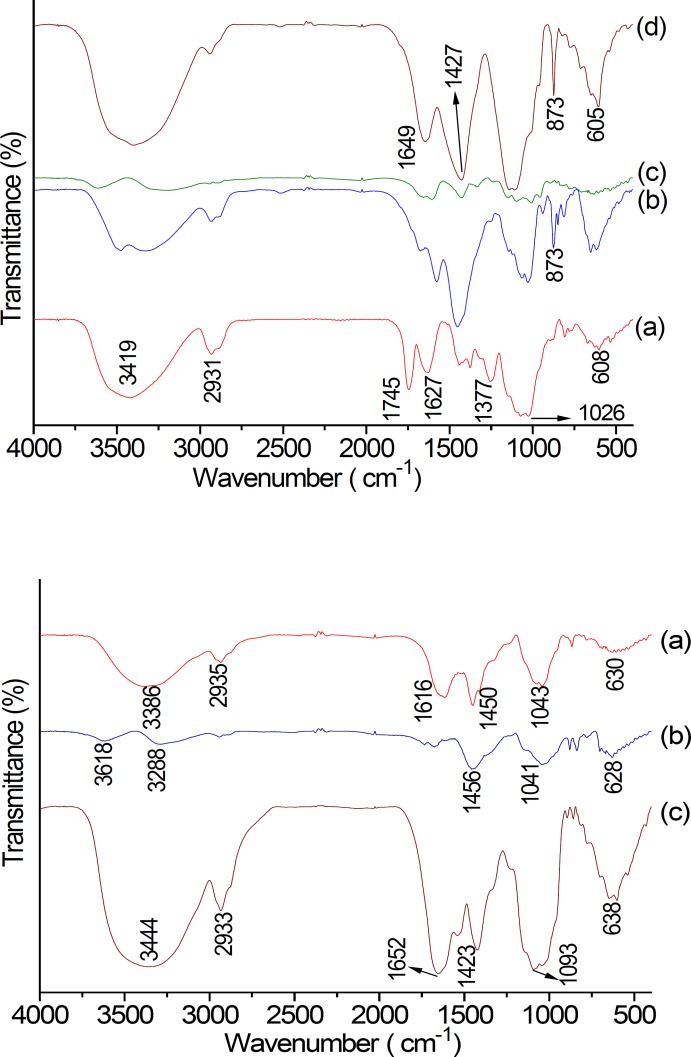
(1) FT**-**IR spectrum of NA fraction: NA(a); NA-1(b); NA-2 (c); NA-3 (d). NA, Neutral Anemaran. (2) FT**-**IR spectrum of AA parts: AA (a), AA-1(b), AA-2 (c).

FT-IR spectrum of NA, AA and their purified fractions are shown in Figure 4-1, 2. The FT-IR spectra of NA and AA were found to be similar. The band between 3600 and 3300 cm^−1^ (NA: 3419 cm^−1^, and AA: 3444 cm^−1^) represented the stretching vibration of polysaccharide glycoside hydroxyl. The small band at around 2933 cm^−1^ was associated with stretching vibration of C–H in the sugar ring (26). The peak at around 1745 cm-1 and the weak one at near 1377 cm^-1^ of NA were different from that of AA. The two peaks above were indicative of the presence of carboxyl groups, which indicated the characteristic FT-IR absorption of uronic acids (27, 28). The bands at 1649 cm^−1^ (Figure 4-1d) and 1652 cm^−1^ (Figure 4-2c) displayed the –CHO stretching vibration or N–H deviational vibration of protein; peak at 1423 cm^-1^ displayed C–O stretching vibration of galacturonic acid. The absorption peak at about 1627 cm^−1^ was due C=O stretch of carboxylic anions (salt) of galacturonic acid. A hydration peak of polysaccharide at 873 cm^-1^ indicated that NA-1 and NA-3 contained the *β*-glycosidic linkage (25, 29). Therefore, *β*-galactan was mainly observed in NA-1 and NA-3. However, there is no absorption at 4000 cm^−1^ and 400 cm^−1^ in NA eluting with 0.3 mol/L NaCl aqueous solution and AA eluting with 0.3 mol/L NaCl aqueous solution. 

*SEM analysis*

**Figure 5:**
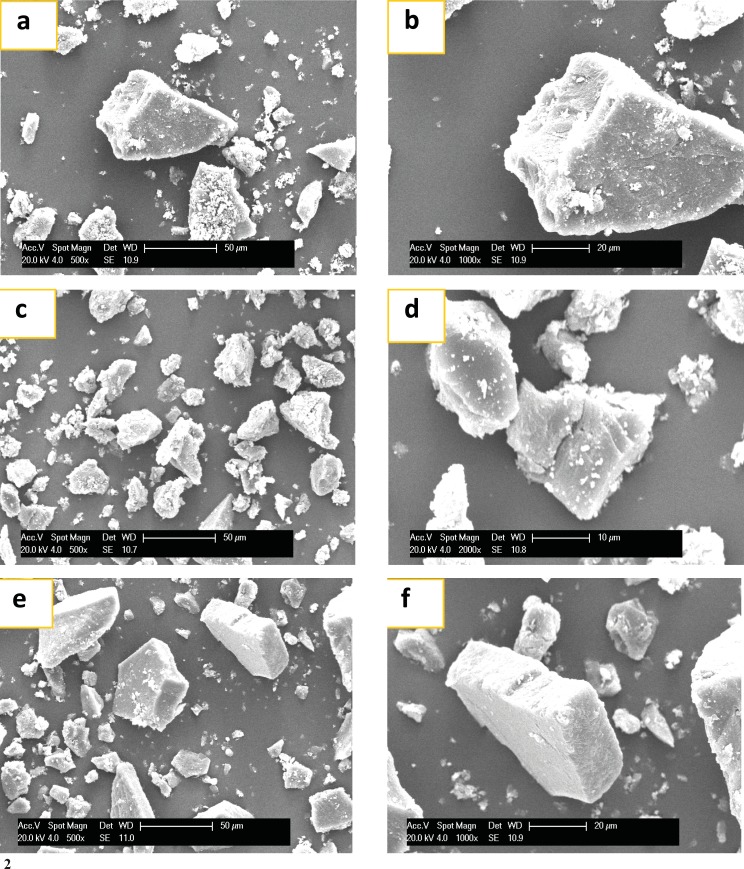
(1) SEM of NA fractions. (a): NA (500×); (b): NA(1000×); (c): NA-1(500×); (d): NA-1(1000×); (e): NA-2 (500×); (f): NA-2 (1000×); (g): NA-3 (500×); (h): NA-3 (1000×). NA: Acid Anemaran. (2) SEM of AA. (a): AA (500×); (b): AA (1000×); (c): AA-1 (500×); (d): AA-1(1000×); (e): AA-2 mol/L (500×); (f): AA-2 (1000×). AA: Acid Anemaran.

The SEM photographs of NA, AA and their different fractions are shown in Figure 5-1, 2. Crude NA anemaran exhibited large irregular non-homogeneous lumps with cracks and particle on the surface of the granules. However, the crude AA displayed polygonal or irregular shape with no cracks. NA-1, NA-2 and NA-3 fractions compared with crude NA showed significant variation in size and shape when viewed by SEM. The size became smaller and fewer particles adhered to the granules with concentrations of NaCl aqueous solution increasing from 0-0.5 mol/L for NA. The similar phenomenon occurred in AA. 

*In-vitro growth inhibition of HepG*_2_ *cells*

The cytotoxicity (growth inhibition) of NA and AA was evaluated in HepG_2_ cells after treatment of the cells with increasing concentrations (0.05, 0.1, 0.5 and 1 μg/mL) of samples using MTT assay against blank control groups (Table 1). With concentration ranging from 0.05 to 0.5 μg/mL, NA did not significantly inhibit the growth of HepG_2 _cells. Furthermore, the effect of NA at concentration of 1μg/mL was not detected. AA did not significantly exhibit inhibition ratios when the concentrations varied from 0.05 to 0.1 μg/mL with inhibition ratio of 0.0027%~0.0192%, which was higher than that of NA. Anemaran and steroidal saponins of Rhizome *Anemarrhena *have been identified as the active components responsible for hypoglycemic action and anti-tumor effects (5-9). However, most studies merely focused on the cytotoxicity of the Rhizome *Anemarrhena *extracts and little information regarding the cytotoxicity. We have successfully identified that the fractions of NA and AA did not significantly inhibit the growth of HepG2 cells.

**Table 1:** Growth inhibition and hypoglycemic effect of two anemarans fractions against HepG2 cells *in-vitro*

**Treatment**	**Concentration ** **(μg/mL)**	**Growth inhibition rate (%)**
Normal Control	−	0
NA	1	nd
	0.5	0.0092 ± 0.0004
	0.1	0.0162 ± 0.0024
	0.05	0.017 ± 0.0018
AA	1	nd
	0.5	nd
	0.1	0.0192 ± 0.0016
	0.05	0.0027 ± 0.0032

nd: not detected

Values are mean ± SD of three replicates. Control cells were incubated with medium alone.

The result was expressed as glucose consumption. ap < 0.01: normal control (without insulin and anemaran treatment); *p < 0.05: Diabetic control (without anemaran treatment).

*Glucose consumption assay*

Complicated metabolic disorders cause diabetes with high blood glucose level. The secretion of insulin may decrease with a decrease in pancreatic *β *cell mass or function disturbances of *β *cells (30). The liver cells are more resistant than the muscle tissue and the fats. Insulin resistance in liver cells results in impaired glycogen synthesis and a failure to suppress glucose production (31). So, we used HepG_2_, a human hepatoma cell line, with high concentration of insulin (2×10^-6^ M) to establish an insulin-resistant cell model, which expressed an insensitive response to normal-concentration insulin (10-9 M). Table 2 showed high-concentration insulin-treated HepG_2_ cells suffered an obvious glucose consumption decrease compared with the normal cells (ap < 0.01), indicating the insulin-resistant model was simulated successfully *in-vitro*. After the liver cells were treated with NA and AA for 24 h, the glucose consumption increased at the concentrations from 0.05 to 1 μg/mL. The results suggest that NA can increase the glucose consumption significantly of insulin-resistant cells compared with the model control cells (*p < 0.05). Insulin-resistant cells were more sensitive to NA than to the AA. However, the purified fractions of NA and AA have no hypoglycemic activity *in-vitro*. It has been reported that anemarans A, B, C and D and many other polysaccharides could decrease of blood glucose *in-vivo *(1). However, the total neutral anemarans showed significant hypoglycemic activity, which is different from the results reported above (1). 

**Table 2:** Hypoglycemic effects of anemarans were evaluated by sensitivity assay of NA and AA to exogenous insulin in insulin-resistant HepG2 cells

**Treatment**	**Concentration (μg/mL)**	**Glucose consumption (mmol/L)**
Normal Control	0	6.82±0.24
Diabetic Control	0	4.35±0.05
NA	1	6.43±0.98*a
	0.5	6.03±0.29*a
	0.1	5.66±1.01*a
	0.05	5.38±0.84
AA	1	5.33±0.87
	0.5	4.73±0.12
	0.1	4.47±0.18
	0.05	4.39±0.13

ap < 0.01: normal control (without insulin and anemaran treatment); *p < 0.05: Diabetic control (without anemaran treatment).

## Conclusions

Previous studies have indicated that anemarans A, B, C and D show hypoglycemic effect. In this study, the isolation process of anemarans was investigated and the optimum isolation condition of extracting anemarans from Rhizome Anemarrhena was obtained. Neutral and acid anemarans nearly did not inhibit growth inhibition of HepG_2_ cells. It was demonstrated that neutral anemarans (NA) exhibited higher hypoglycemic effect than acid anemarans (AA). According to our purification experiment, five different polysaccharide fractions, NA-1, NA-2, NA-3, AA-1 and AA-2, were obtained. SEM result showed that neutral fractions, NA-1 and AA-1 have smaller particles adhered to the granule surface than acid fractions. According to FT-IR, the peaks at about 873 cm^-1^ revealed *β*-galactan mainly observed in NA-1 and NA-3. Further investigations need to be carried out to check the activity of each fraction of purified anemaran. 
